# Genetic biomarkers predict response to dual BCL-2 and MCL-1 targeting in acute myeloid leukaemia cells

**DOI:** 10.18632/oncotarget.26540

**Published:** 2018-12-28

**Authors:** Martin Grundy, Sahana Balakrishnan, Matthew Fox, Claire H. Seedhouse, Nigel H. Russell

**Affiliations:** ^1^ Clinical Haematology, Nottingham University Hospitals, Nottingham, United Kingdom; ^2^ Department of Haematology, Division of Cancer and Stem Cells, University of Nottingham, Nottingham, United Kingdom

**Keywords:** AML, BCL-2, MCL-1, Venetoclax, S63845

## Abstract

Acute myeloid leukaemia (AML) cells often up-regulate pro-survival members of the BCL-2 protein family, such as BCL-2 and MCL-1, to avoid apoptosis. Venetoclax (ABT-199) targets BCL-2 and has shown promising efficacy in AML but over-expression of MCL-1 can cause resistance. A co-operative approach, targeting both BCL-2 and MCL-1 may therefore prove beneficial. This study investigated the potential synergistic relationship between Venetoclax and the MCL-1 inhibitor S63845 in AML cells. We treated MV4-11 cells and primary AML samples for 4 hours with Venetoclax, S63845 or the combination. We used a short-term flow cytometric technique to assess synergy using cytochrome C release as a read out of response. The combination of Venetoclax and S63845 produced a synergistic apoptotic response in MV4-11 cells and primary samples, including the leukaemia re-populating leukaemic stem cell (LSC) population, in 92% of the samples. Known molecular biomarkers of response to BCL-2 and MCL-1 targeting agents were corroborated, and augmented, with the short-term functional assay. The assay also predicted potential biomarkers of response to the combination of BCL-2 and MCL-1 targeting agents. Primary samples with an IDH2_140 mutation were more sensitive to Venetoclax as a single agent whereas samples with a FLT3-ITD mutation were more resistant. This resistance could be reversed when combined with S63845. All FLT3-ITD and NPM1 mutated samples were sensitive to the combination of drugs.

We report that co-operatively targeting BCL-2 and MCL-1 may be beneficial in AML and a short-term *in vitro* assay can identify patients who might best respond to this combination.

## INTRODUCTION

AML is a malignant clonal disorder of the progenitor cells in the bone marrow. The heterogeneity of the disease and of patient related factors makes prognosis and predicting responses to treatment difficult. Despite a substantial increase in the understanding of the pathophysiology, cytogenetics and molecular genetics of AML, treatment options have advanced only slowly over the past 40 years [1]. In 2017 however, four drugs received US Food and Drug Administration (FDA) approval for treating AML. These include midostaurin and enasidenib targeting mutant FLT3 and IDH2 respectively, the liposomal cytarabine-daunorubicin formulation CPX-351, and the re-approval of the CD33 targeting antibody gemtuzumab ozogomycin [2]. The emergence of BH3-mimetics designed to target the anti-apoptotic members of the BCL-2 (B-cell lymphoma-2) family has provided another exciting therapeutic option. The apoptotic fate of a cell depends on a fine balance of interactions between pro-survival molecules such as BCL-2, MCL-1 (Myeloid Cell Leukaemia-1) and BCL-X_L_ (B cell lymphoma-extra-large) and BH3-only protein sensitizer molecules such as BAD (BCL-2 associated death promotor) and NOXA (PMAIP1; Phorbol-12myristate-13-acetate-induced protein 1) [3]. Following a cell death stimulus, BH3-only sensitizer proteins are activated, and displace BH3-only activator proteins such as BIM (BCL-2 interacting mediator of cell death) and BID (BH3 interacting-domain death agonist) from their pro-survival molecular chaperones, resulting in activation of effector molecules BAX (BCL-2 associated X protein) and BAK (BCL-2 homologous antagonist killer). BAX and BAK subsequently oligomerise and form pores that cause mitochondrial outer membrane permeabilisation resulting in cytochrome C release and apoptosis.

Overexpression of BCL-2 is associated with tumour progression and resistance to chemotherapy in multiple malignancies, including AML [4, 5]. The most promising BCL-2 inhibitor to date is the BH3 mimetic Venetoclax, which has demonstrated clinical promise in AML, particularly when used in combination with cytarabine or hypomethylating agents [6, 7]. Dormant LSCs can repopulate a leukaemia following treatment, resulting in relapse, and a defining characteristic of these cells is high levels of BCL-2 [8]. We have previously demonstrated that these leukaemia re-populating cells are sensitive to Venetoclax treatment [9]. MCL-1 is also a crucial pro-survival factor in AML [10]. We have previously reported that a number of agents that indirectly inhibit MCL-1 expression produce a synergistic apoptotic response when used in combination with Venetoclax in AML cells [11, 12]. Resistance to Venetoclax monotherapy is associated with released BIM being sequestered by MCL-1 and others have reported that this can be reversed by combining Venetoclax with cytarabine or daunorubicin [13]. Until now a truly selective and potent small molecule inhibitor of MCL-1 has not been developed and a worrying caveat is that genetic deletion of MCL-1 in mouse models resulted in bone marrow failure and myocardial toxicity [14]. It is hoped that transient inhibition of MCL-1 using a BH3-mimetic drug could circumvent these side effects. S63845 is a novel MCL-1 targeting BH3-mimetic, developed by Servier, that has shown low toxicity in pre-clinical models and has demonstrated syergy in combination with Venetoclax in T-cell acute lymphoblastic leukaemia [15–17].

Predictive assays to demonstrate which drugs an individual patient will best respond to are still lacking. An issue that limits traditional chemosensitivity assays is that AML cells are fragile *ex vivo*, and most will die spontaneously in culture fairly rapidly [18, 19]. By focusing on a same-day functional assay, with intact cells, we aim to overcome this obstacle. Using this assay, we demonstrate that co-operative targeting of BCL-2 and MCL-1 with the BH3-mimetics Venetoclax and S63845 induces an enhanced apoptotic response in bulk and LSC populations of primary AML samples. Known molecular biomarkers of response to single agents were corroborated using the assay. The assay also augmented our knowledge of current biomarkers along with predicting potential biomarkers of response to the combination of BCL-2 and MCL-1 targeting agents.

## RESULTS

### Co-operative dynamic BH3 profiling

**Figure 1:**
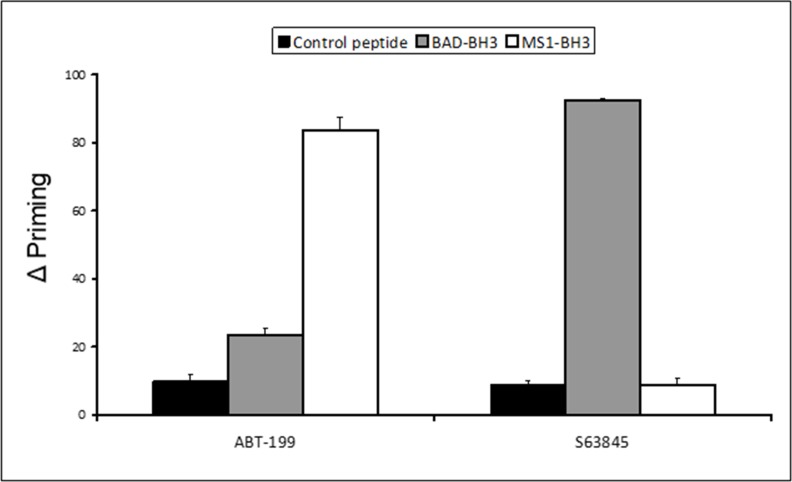
Co-operative dynamic BH3 profiling assay: delta priming to BAD-BH3 and MS1-BH3 peptides Delta priming in MV4-11 cells is measured by cytochrome C release after 4 hours Venetoclax (50 nM) S63845 (5 nM) drug treatment and additional incubation with the indicated BH3 peptides (BAD-BH3 at 3 μM, MS1-BH3 at 3 μM, PUMA2A control at 100 μM). Values are corrected for cytochrome C release with peptide only as described in the methods. Columns, mean of three experiments; bars, SD (*n* = 3).

Dynamic BH3 profiling is a technique that involves priming mitochondria following short-term drug exposure and measuring subsequent BH3 peptide-induced cytochrome C release [20]. Using the technique we have previously shown that the BCL-2 antagonist Venetoclax sensitises to the MCL-1 inhibitory MS1-BH3 peptide, whilst agents that target MCL-1 non-specifically, sensitise to the BCL-2 inhibitory BAD-BH3 peptide [11]. However, despite exhibiting synergy with Venetoclax, the specific MCL-1 binding inhibitor A-1210477 failed to prime to BAD-BH3 at sub-micromolar concentrations. It was therefore important to test the credentials of S63845 as a BH3-mimetic in this system. We used MV4-11 cells in this assay as they are sensitive to BCL-2 and MCL-1 targeting agents but they do not over-express BCL-X_L_ [12]. We used the synthetic peptide MS1 as it binds to MCL-1 with higher affinity than NOXA-BH3 [21]. Venetoclax was included as an experimental control and priming to MS1-BH3 peptide was confirmed (Figure 1). S63845 primed to BAD-BH3 peptide but not to MS1-BH3 peptide confirming its credentials as a BH3-mimetic and suggesting its co-operative potential with Venetoclax. The concentrations of Venetoclax and S63845 used in the experiment were determined as previously reported [11].

### Co-operative induction of apoptosis with the combination of Venetoclax and S63845 in MV4-11 cells

**Figure 2:**
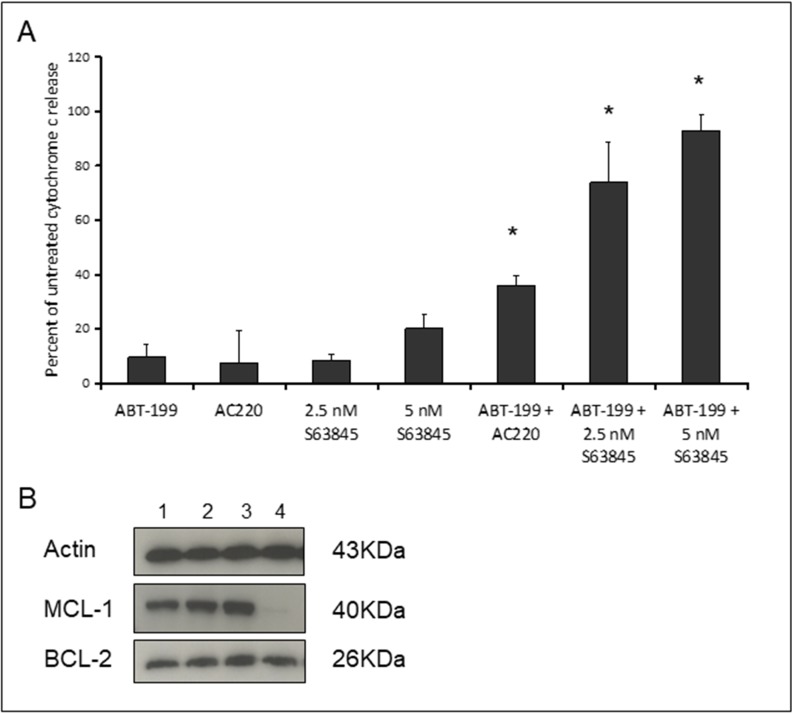
Co-operative induction of apoptosis with the combination of Venetoclax and S63845 (**A**) MV4-11 cells were treated with 10 nM Venetoclax, 10 nM AC220, 2.5 nM or 5 nM S63845 or the indicated combinations. After 4 hours, cells were fixed and processed for cytochrome C release. Columns, mean of three experiments; bars, SD (*n* = 3). Asterisks indicate synergy according to the Bliss Independence model as described in the methods. (**B**) MCL-1 and BCL-2 protein was quantified in untreated MV4-11 cells (Lane 1) or cells treated for four hours with 10 nM Venetoclax (Lane 2), 5 nM S63845 (Lane 3) or the drug combination (Lane 4). The blots shown are cropped and an example of two independent experiments.

MV4-11 cells were treated for four hours with Venetoclax and S63845 alone or in combination followed by measurement of cytochrome C release (Figure 2A). The FLT3 inhibitor AC220 was included as an experimental control as we have previously demonstrated its co-operative induction of apoptosis in MV4-11 cells in combination with Venetoclax [11]. Dose response assays were performed to select the drug concentration that produced 0-20% cytochrome C release as a single agent. A strong synergistic apoptotic response was observed with the combination of Venetoclax and S63845 at low nanomolar concentrations. We have previously reported apoptotic synergy with Venetoclax using various drugs that target MCL-1 non-specifically. All of these non-specific agents caused MCL-1 protein degradation as single agents after four hours [11]. Here we show that there is no reduction of MCL-1 protein expression when S63845 is used as a single agent (Figure 2B). The combination of S63845 and Venetoclax results in loss of MCL-1 protein whilst BCL-2 expression remains unchanged. See Supplementary Figure 1 for uncropped blots.

### Co-operative induction of apoptosis with the combination of Venetoclax and S63845 in primary AML samples

**Figure 3:**
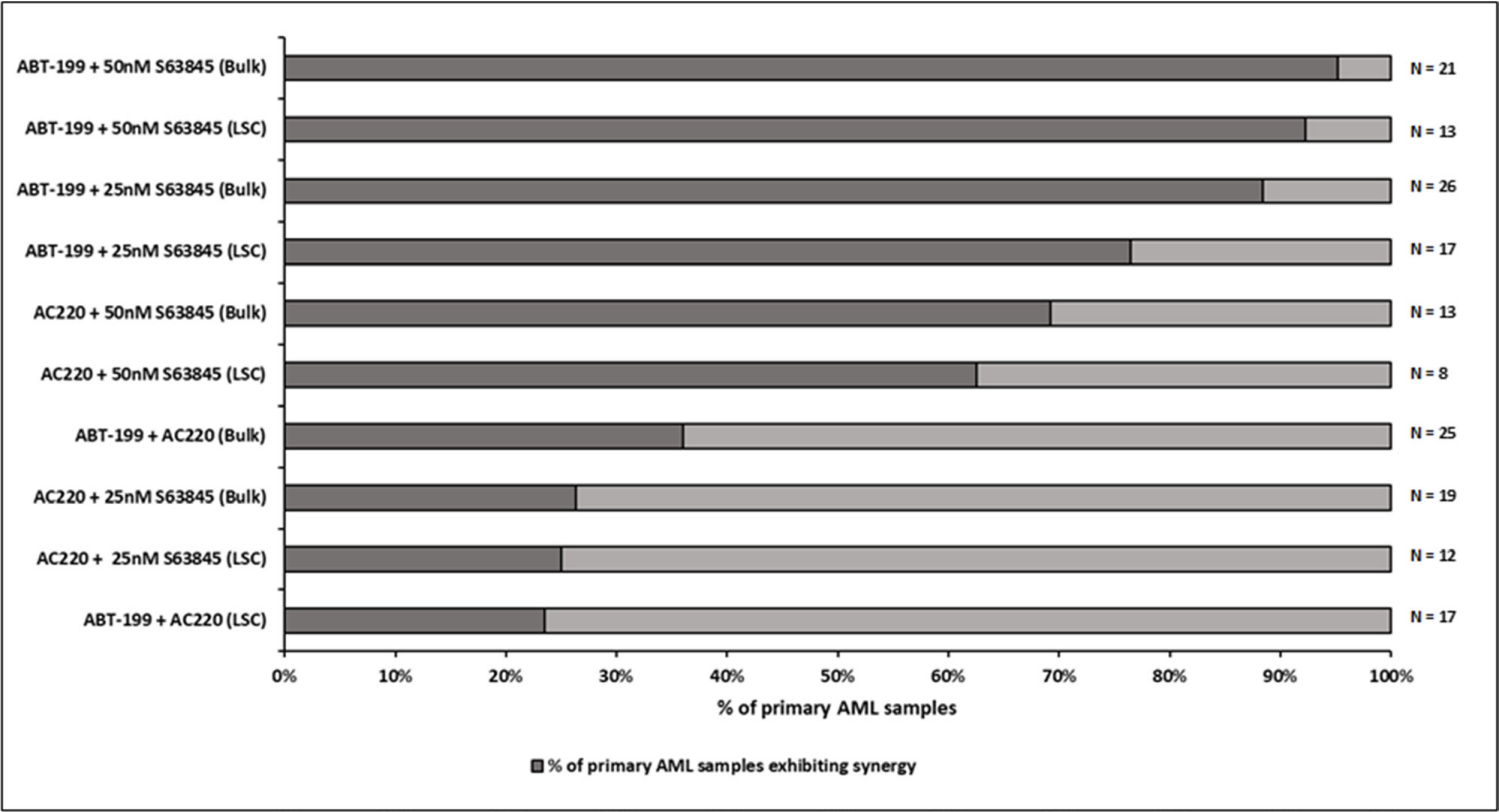
Co-operative induction of apoptosis with the combination of Venetoclax and S63845 in primary AML samples–Summary of drug combination response Primary AML cells were treated with single agent or the indicated combinations of Venetoclax (100 nM), AC220 (100 nM) and S63845 (25 nM or 50 nM). After 4 hours, cells were fixed and processed for cytochrome C release. The LSC (leukaemic stem cell) population was distinguished from the bulk population by CD34/CD38 staining. The darker part of the bar demonstrates the percentage of primary samples exhibiting synergy for a particular drug combination. Synergy was calculated according to the Bliss Independence model as described in the methods.

**Figure 4:**
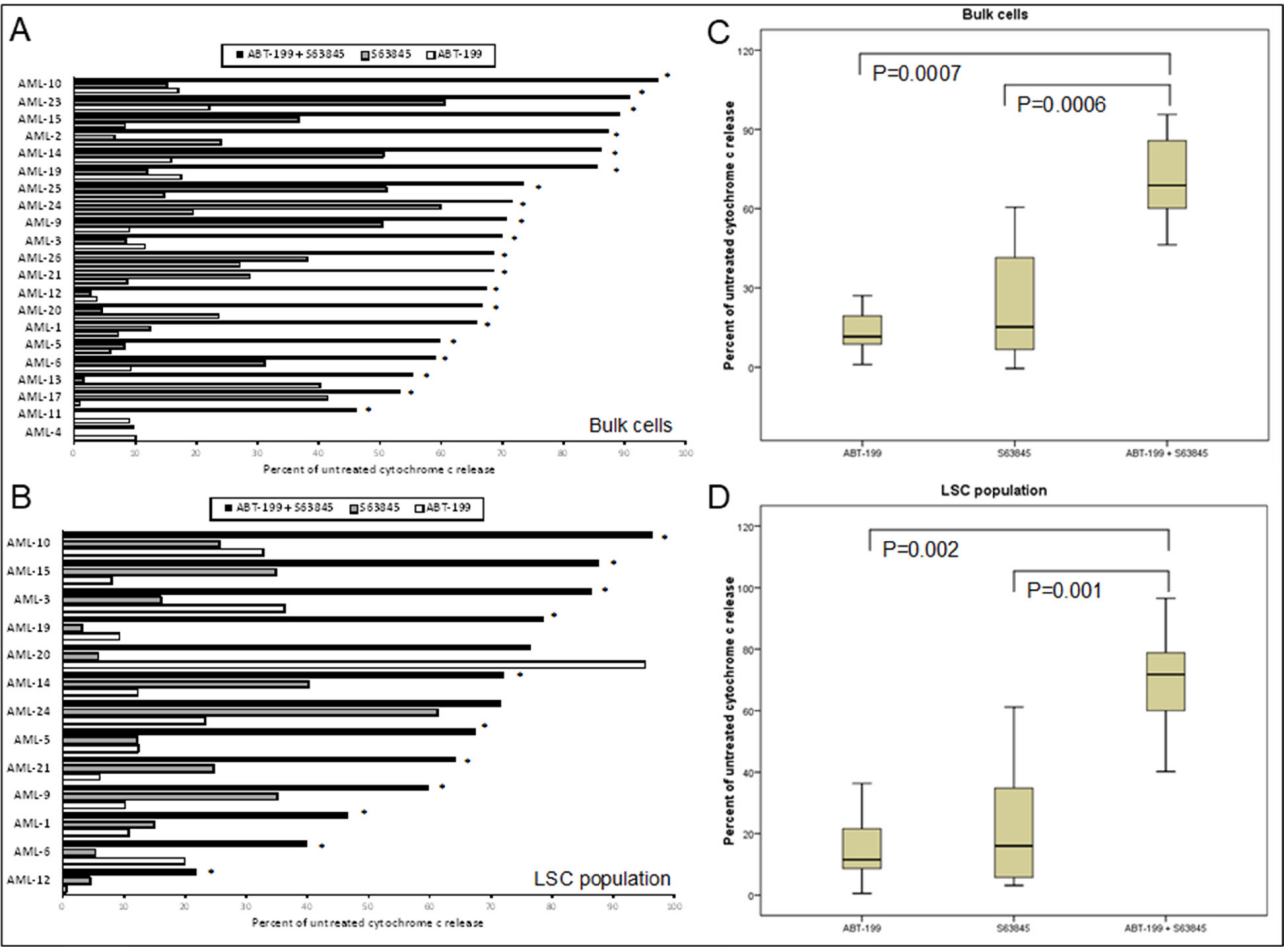
Co-operative induction of apoptosis with the combination of Venetoclax and S63845 in primary AML samples Primary AML cells were treated with 100 nM Venetoclax or 50 nM S63845 or a combination of both. After 4 hours, cells were fixed and processed for cytochrome C release. Cytochrome C release is shown in the bulk population of cells (**A**) (*n* = 21) and the LSC population of cells (**B**) (*n* = 13). Asterisks indicates synergy according to the Bliss Independence model as described in the methods. Summarising the median interquartile range (boxes) and range (error bars) of cytochrome C release in the bulk population of 21 primary AML samples (**C**) and the LSC population of 13 samples (**D**).

Primary AML samples were treated for four hours with Venetoclax, S63845 and AC220 alone, or in combination followed by measurement of cytochrome C release (Figure 3). Experience with the assay has taught us that using 10x the drug concentration used in cell lines should give us the range of cytochrome C release with single agents that allows us to detect any synergy in primary samples. The FLT3 inhibitor AC220 was included as an experimental control as when used as a single agent we would expect it to specifically target samples bearing a high FLT3-ITD mutant allelic burden [22]. This would indicate whether this assay was a good predictor of primary samples response to drugs. Interestingly the combination of AC220 and S63845 resulted in >60% synergistic response in primary sample bulk and LSC populations. By far the most striking synergy resulted from the combination of Venetoclax and S63845 with 20 out 21 (95%) samples (Bulk population) and 12 out of 13 (92%) samples (LSC population) showing synergy using the higher dose of S63845. In the only sample, (AML-20) where synergy was not seen in the LSC population, this was due to a maximal response to Venetoclax as a single agent. Figure 4A (Bulk cells) and 4B (LSC) show raw data for primary samples treated with the combination that gave the best response as shown in Figure 3 (100 nM Venetoclax or 50 nM S63845 alone or a combination of both) along with statistical analysis (Figure 4C and 4D).

### A short-term cytochrome C release assay predicts primary sample response to drugs

**Figure 5:**
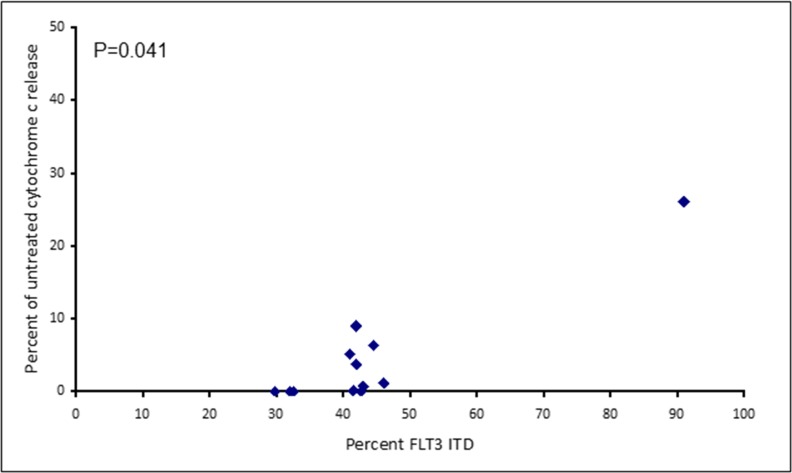
Apoptotic response to AC220 in primary AML samples correlates to FLT3 ITD allelic burden Primary AML cells were treated with 100 nM AC220 for 4 hours. Cells were then fixed and processed for cytochrome C release.

**Figure 6:**
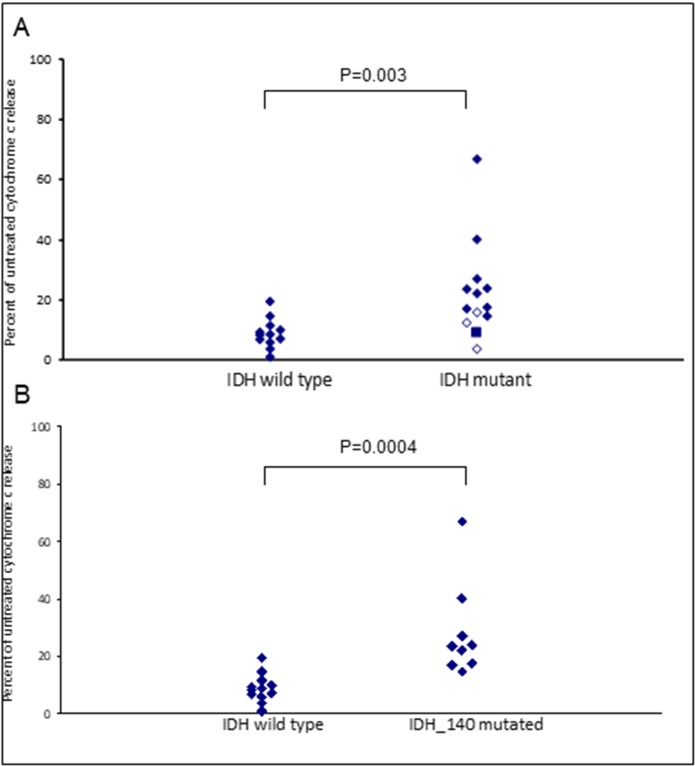
IDH mutational status in primary AML samples predicts apoptotic response to Venetoclax Primary AML cells were treated with 100 nM Venetoclax for 4 hours. Cells were then fixed and processed for cytochrome C release. Samples are grouped as either wild-type or mutant according to IDH2_140 (filled diamonds), IDH1_132 (closed diamonds) or IDH2_172 (square) mutational status (**A**) or IDH2_140 mutational status (**B**).

**Figure 7:**
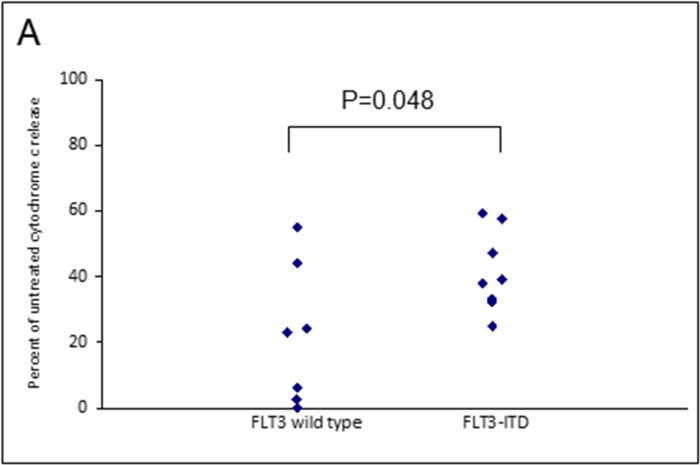
FLT3 mutational status in primary AML samples predicts apoptotic response to MCL-1 targeting agents Primary AML cells were treated with the combination of 50 nM S63845 and 100 nM AC220 for 4 hours. Cells were then fixed and processed for cytochrome C release and grouped as either wild-type or mutant according to FLT3 mutational status.

**Figure 8:**
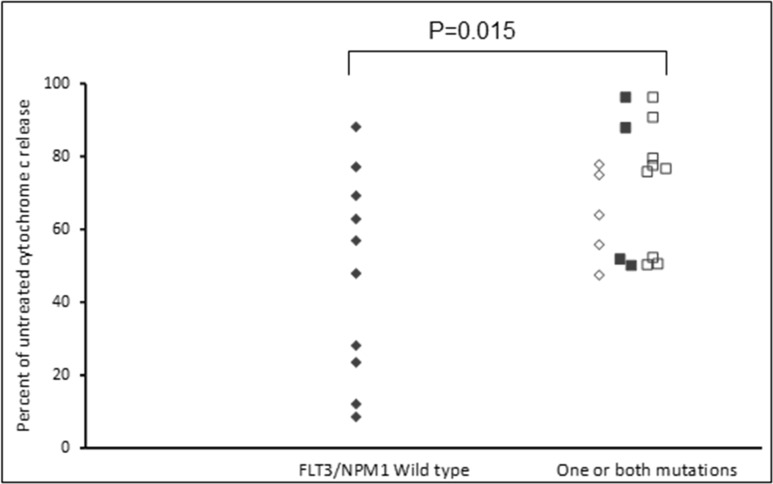
FLT3 and NPM1 mutated primary AML samples are sensitive to the combination of Venetoclax and S63845 Primary AML samples were treated with a combination of 100 nM Venetoclax and 50 nM S63845 for 4 hours. Cells were then fixed and processed for cytochrome C release. Samples are grouped as FLT3-/NPM1- (filled diamonds), FLT3+/NPM1- (open diamonds), FLT3-/NPM1+ (filled squares) or FLT3+/NPM1+ (open squares).

**Figure 9:**
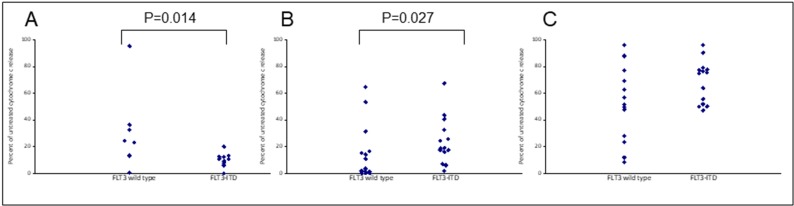
Apoptotic resistance to Venetoclax in FLT3 mutated primary samples can be reversed when combined with S63845 Primary AML cells were treated with 100 nM Venetoclax (**A**), 50 nM S63845 (**B**) or a combination of both (**C**) for 4 hours. Cells were then fixed and processed for cytochrome C release. Samples are grouped as either wild-type or mutant according to FLT3 mutational status.

Assays that predict what drug an individual AML patient will best respond to are still lacking. An issue is that primary AML cells often survive poorly *in vitro*, thus confounding conventional cytotoxicity assays [19]. We have previously demonstrated two short-term flow cytometric assays that predict long term chemo-responsiveness to drugs in AML cell lines [23]. We investigated whether a four hour cytochrome C release assay could confirm drug response to known molecular biomarkers and predict other markers of response. Having a FLT3-ITD mutation does not necessarily predict response to FLT3 inhibitors, particularly *in vitro*. The FLT3-ITD allelic burden appears a better predictor of response [22]. Our data corroborates this in that the amount of cytochrome C release after four hours of AC220 treatment correlated with the FLT3-ITD allelic burden (*p* = 0.041) indicating the assay could be a useful predictor of drug response (Figure 5). The most striking correlation resulting from our data was the impact of isocitrate dehydrogenase (IDH) mutational status on the sensitivity to Venetoclax as a single agent (*p* = 0.003) (Figure 6A). This agrees with early clinical data that has indicated that patients with an IDH1/2 mutation could preferentially benefit from Venetoclax therapy [24, 25]. Our data suggetsts that patients with an IDH2_140 mutation might specifically benefit. The more common IDH2_140 mutated samples were the most responsive to Venetoclax when compared to wild-type samples (*p* = 0.0004) (Figure 6B). One possibly unexpected result was the synergy seen in primary samples with the combination of the FLT3 inhibitor AC220 and the MCL-1 targeting S63845 (Figure 3). Mutation of FLT3 results in up-regulation of the pro-survival protein MCL-1 through STAT5 activation [26]. This combination of drugs is therefore targeting MCL-1 through two separate mechanisms and the FLT3-ITD primary samples (*p* = 0.048) (Figure 7) responded best to this combination. All FLT3-ITD and NPM1 mutated samples were sensitive to cytochrome C release when treated with the combination of Venetoclax and S63845 (*p* = 0.015) (Figure 8). Also of note was that the FLT3-ITD samples were relatively resistant to single agent Venetoclax when compared to wild-type samples (*p* = 0.014) (Figure 9A). Conversely, FLT3-ITD samples were more sensitive to S63845 as a single agent (*p* = 0.027) (Figure 9B). FLT3-ITD sample resistance to Venetoclax could be reversed when combined with S63845 (Figure 9C).

## DISCUSSION

Our inability to target the leukaemia re-populating LSCs with conventional chemotherapy is a confounding issue in the progress of AML therapy. A major finding in recent years has been the identification of high levels of BCL-2 as a defining characteristic of LSCs [8]. Venetoclax is a clinically established BCL-2 targeting BH3-mimetic that has FDA approval for use in CLL patients with a 17p deletion [27, 28]. In November 2018, Venetoclax gained FDA approval for use in combination with low-dose cytarabine or hypomethylating agents, in the treatment of AML. A phase II Venetoclax monotherapy study in older patients who had many high-risk features resulted in a promising 19% complete morphologic response [24]. However, despite their initial response, all patients treated with Venetoclax eventually relapsed, suggesting a resistance mechanism to Venetoclax monotherapy. This prompted clinical trials using Venetoclax in combination with other drugs in AML and impressive clinical responses, particularly when used in combination with cytarabine or hypomethylating agents, have already been documented [7, 29]. Resistance to Venetoclax monotherapy is associated with pro-apoptotic BIM, being released from BCL-2, which is then sequestered by over-expressed MCL-1 or BCL-X_L_ anti-apoptotic proteins [30]. MCL-1 is the preferred target, as agents such as Navitoclax that target both BCL-2 and BCL-X_L,_ have resulted in platelet toxicity and dose limiting thrombocytopenia [31]. When used in combination with Venetoclax, cytarabine results in decreased MCL-1 protein levels, decreased association of MCL-1 with BIM, and synergistic induction of cell death in AML cells [13]. By simultaneously targeting MCL-1, resistance to Venetoclax may be avoided, and we and other groups have reported that dual targeting of BCL-2 and MCL-1 in AML produces an enhanced cell killing response [11, 32–34]. Worryingly though, it has been reported that simultaneous inhibition of BCL-2 and MCL-1, synergistically inhibits the proliferation of peripheral blood mononuclear cells as well as AML cells [32]. In addition, deletion of MCL-1 in mouse models resulted in bone marrow failure and myocardial toxicity [14]. It is hoped that these problems can be avoided by transiently targeting MCL-1 with a BH3-mimetic and importantly simultaneous targeting of BCL-2 and MCL-1 with BH3-mimetics resulted in minimal toxicity to normal haematopoietic progenitor cells [33]. S63845 is a novel MCL-1 targeting BH3-mimetic that has demonstrated low toxicity in pre-clinical models and kills AML cells as a single agent [15]. We previously reported that although the specific MCL-1 binding inhibitor A-1210477 produced a supra-additive effect in combination with Venetoclax in AML cells, it failed to prime to BAD-BH3 peptide in a BH3 profiling assay [11]. We confirmed here using BH3 profiling that S63845 does prime to BAD-BH3 peptide and is thus acting at the mitochondria as a bona fide BH3-mimetic. We have also previously reported drugs that indirectly target MCL-1, when used in combination with Venetoclax, produce a synergistic apoptotic response [11, 12]. All of these MCL-1 targeting drugs resulted in MCL-1 protein depletion as single agents. We confirm here that S63845 does not result in loss of MCL-1 protein as a single agent suggesting its different and potentially clinically beneficial mechanism of action. The combination of S63845 and Venetoclax resulted in MCL-1 depletion whilst BCL-2 expression remained unchanged. This difference is likely reflected by the very short half-life of MCL-1 (approximately one hour) when compared to BCL-2 (10–14 hours) [35, 36].

Here we also report a same day functional assay, using primary AML samples, which demonstrates the co-operative potential of targeting both BCL-2 and MCL-1, along with identifying molecular biomarkers of response. This assay could offer clinicians a faster and more targeted therapeutic window when compared to mouse PDX models and colony assays. The short time frame of the assay is important as AML cells are particularly fragile *in vitro*, and the majority will die spontaneously in culture fairly rapidly [19]. We saw a significant co-operative response using the combination of Venetoclax and S63845 in MV4-11 cells and primary samples. The synergistic response seen in primary samples was remarkable, particularly considering the short time frame of the assay, and was much greater than we have previously reported with agents that target MCL-1 indirectly [12]. Importantly, this response was also seen in the leukaemia re-populating LSC populations of primary samples. LSCs are the most difficult cell subgroup to therapeutically target in AML and are usually the source of resistance and relapse [37]. The dual targeting of BCL-2 and MCL-1 therefore offers the exciting potential of achieving deeper levels of sustainable remission in AML patients.

Along with being a short-term readout of response, the cytochrome C release assay was able to confirm known molecular markers of response to single agents, and predict biomarkers to the drug combination. The assays validity of being a useful predictor of drug response was demonstrated, as the amount of cytochrome C release following treatment with the FLT3 inhibitor AC220, correlated with the amount of FLT3-ITD mutant allelic burden in the primary samples [22]. The assay was further validated with the sensitivity of IDH mutated samples to Venetoclax as a single agent. IDH mutations are known to induce BCL-2 dependence and early clinical data has indicated that patients with an IDH1/2 mutation could preferentially benefit from Venetoclax therapy [24, 25, 38]. Here we report for the first time that patients with the more common IDH2_140 mutation might specifically benefit. The samples we tested with the less common IDH1_132 or IDH2_172 mutations were less sensitive to Venetoclax than those with an IDH2_140 mutation were. All three IDH mutations result in elevated levels of the oncometabolite 2-hydroxyglutarate (2-HG), although the mutations are not functionally equivalent, and a better prognosis for patients with IDH2_140 mutations *versus* those with IDH1_132 or IDH2_172 mutations has been reported by multiple groups [39–41]. 2-HG accumulation leads to inhibition of cytochrome C oxidase and disruption of the mitochondrial electron transport chain, lowering the apoptotic threshold of cells, making them dependent on BCL-2 for survival [38]. An IDH2_172 mutation leads to greater accumulation of 2-HG than does an IDH2_140 mutation suggesting a less severe phenotype in the IDH2_140 mutated cells and potentially explaining the differences in sensitivities to Venetoclax [42].

There was only one primary sample LSC population where no drug synergy was observed with the combination of Venetoclax and S63845 and this was because the sample was particularly sensitive to Venetoclax as a single agent. Unsurprisingly, this sample had an IDH_140 mutation, and on further investigation, it was also found to harbour a DNMT3A mutation. DNMT3A mutations have been associated with activated RAS kinase signalling and subsequent overexpression of BCL-2 [43, 44]. Further research is required to examine if patients with this combination of mutations might preferentially benefit from Venetoclax treatment.

FLT3-ITD primary samples were more sensitive to S63845 as a single agent and this might be expected as mutation of the FLT3 receptor results in up-regulation of MCL-1 through STAT5 activation [26]. Conversely, FLT3 mutated samples were less sensitive to Venetoclax, but this was reversed with the addition of S63845. Notably, all primary samples that had a FLT3 or NPM1 mutation were sensitive to the combination of Venetoclax and S63845. FLT3 mutated primary samples were also particularly sensitive to the combination of S63845 and the FLT3 inhibitor AC220. MCL-1 is up regulated in FLT3 mutated cells through STAT5 activation [26]. This suggests that FLT3-ITD samples can be sensitised by targeting MCL-1 through two separate mechanisms. Other groups have reported TP53 mutated samples to be sensitive to Venetoclax whilst others report the drugs mechanism of action to be independent of TP53 status [6, 45]. All of our primary samples were TP53 wild-type so we cannot add to this debate. Importantly, the molecular markers of response identified using the cytochrome C release assay, correlate with early clinical findings using Venetoclax. A phase 1/2 study that combined Venetoclax with low-dose cytarabine reported NPM1, FLT3-ITD, DNMT3A and IDH1/2 mutations as possible biomarkers of response [25].

In conclusion, co-operative targeting of BCL-2 and MCL-1 results in synergistic apoptosis in primary AML cells including the LSC population. We report a real-time cytochrome C release assay which confirms known biomarkers of response to drugs and identifies others. The assay has the potential to determine which patient sub-groups are likely to respond to novel agents in the future. The assay could be particularly useful in predicting response to agents that directly target anti-apoptotic BCL-2 proteins, such as BH3-mimetics.

## MATERIALS AND METHODS

### Reagents

Drugs and reagent suppliers used in the study were as follows: Venetoclax (ABT-199) was supplied by Bioquote limited, York, UK, and S63845 (Active Biochem, Kowloon, Hong Kong), AC220 (Stratech Scientific Ltd, Ely, UK). BIM, BAD, MS1 and PUMA2A BH3 peptides were supplied by Genscript (Piscataway, NJ, USA). All other reagents were from Sigma (Poole, Dorset, UK) unless specified.

### Primary samples

The investigation was conducted on samples obtained with informed consent in accordance with the ethical standards and according to the Declaration of Helsinki and according to national and international guidelines and has been approved by the authors’ institutional review board. Mononuclear cells were obtained by standard methods from bone marrow or peripheral blood samples of patients with AML and cells were cryopreserved until use. Only samples with >90% post-thaw viability were assayed. FLT3 and NPM mutations were determined as reported previously [46]. The majority of p53 mutations occur in exons 5–9 and therefore only these exons were assessed. Screening for p53 and IDH1/2 and mutations was via high resolution melting curve analysis with MeltDoctor HRM mastermix (Thermo Fisher Scientific, Loughborough UK) and performed on a 7500 Fast Real Time PCR system (Thermo Fisher Scientific) according to the manufacturer's instructions. Supplementary Table 1 contains the primer sequences. All mutated and 10% of samples assigned wild-type by HRM were verified by sequencing. All primary samples tested were p53 wild-type. Patient sample demographics are listed in Table 1.

**Table 1:** Characteristics of primary AML patient samples

Sample ID	Gender	Age (years)	Disease Status	Genetic mutations(s)	% FLT3-ITD
AML-1	Male	38	Newly diagnosed	NPM1, FLT3-ITD	32
AML-2	Female	64	Newly diagnosed	NPM1, FLT3-ITD, IDH2_140	30
AML-3	Male	71	Newly diagnosed	NPM1	-
AML-4	Male	73	Newly diagnosed	-	-
AML-5	Female	51	Newly diagnosed	NPM1, FLT3-ITD	42
AML-6	Male	57	Newly diagnosed	FLT3-ITD	91
AML-7	Male	76	Newly diagnosed	-	-
AML-8	Male	20	Newly diagnosed	FLT3-ITD	46
AML-9	Female	70	Newly diagnosed	FLT3-ITD	42
AML-10	Male	37	Newly diagnosed	NPM1, IDH2_140	-
AML-11	Female	51	Newly diagnosed	IDH2_172	-
AML-12	Female	48	Newly diagnosed	IDH1_132	-
AML-13	Male	70	Newly diagnosed	IDH2_140	-
AML-14	Male	79	Newly diagnosed	NPM1, FLT3-ITD, IDH1_132	45
AML-15	Female	83	Newly diagnosed	NPM1, FLT3-ITD	43
AML-16	Male	78	Newly diagnosed	NPM1, IDH2_140	-
AML-17	Female	35	Newly diagnosed	NPM1	-
AML-18	Male	68	Newly diagnosed	IDH2_140	-
AML-19	Male	41	Newly diagnosed	FLT3-ITD, IDH2_140	32.6
AML-20	Female	65	Newly diagnosed	IDH2_140, DNMT3aǂ	-
AML-21	Female	60	Newly diagnosed	FLT3-ITD	21.4
AML-22	Male	78	Relapsed	IDH1_132	-
AML-23	Male	68	Newly diagnosed	IDH2_140	-
AML-24	Female	66	Newly diagnosed	-	-
AML-25	Male	76	Newly diagnosed	NPM1, FLT3-ITD	42
AML-26	Male	67	Newly diagnosed	NPM1, FLT3-ITD, IDH2_140	41

^ǂ^This was the only sample sequenced for a DNMT3a mutation.

### Cells

MV4-11 cells were obtained from the American Type Culture Collection (Manassas, VA, USA) and maintained in RPMI 1640 medium with 10% foetal calf serum (FCS; First Link, Birmingham, UK), 2 mM L-glutamine, 100 U/ml penicillin and 10μg/ml streptomycin. Cultures were sustained at 37°C in 5% CO_2_ and all experiments were performed with cell lines in log phase. Regular testing to authenticate these cells was performed using multiplex short tandem repeat analysis (Powerplex 16, Promega, Southampton, UK). Mycoplasma testing was routinely performed using the Mycoalert mycoplasma detection kit (Lonza, Rockland, USA) and following the manufacturer's instructions.

### Dynamic BH3 profiling

MV4-11 cells were incubated at 5 × 10^5^/ml in culture medium for four hours with the indicated drug. Cytochrome c release (using Alexa-647-conjugated cytochrome c antibody, Becton Dickinson) was measured after a further 60 minute incubation of digitonin permeabilised cells with BH3 peptides as described [12, 47]. Adjustments for peptide-induced cytochrome c release in untreated cells were made in order to establish agent-specific release (delta priming), using the formula 100X (percent cytochrome c positive with peptide – percent cytochrome c positive with drug plus peptide)/(percent cytochrome c positive with peptide). A mutated PUMA-BH3 peptide (PUMA2A) (Ryan *et al.* 2013) at 100 μM and BIM-BH3 peptide (10 μM) were used as controls in all experiments. Data were collected on a FACSCanto II flow cytometer (Becton Dickinson) and analysed with FACS Diva software (Becton Dickinson).

### Cytochrome C release assay

MV4-11 cells were incubated at 5 × 10^5^/ml in culture medium for four hours with Venetoclax (10 nM), AC220 (10 nM) or S63845 (2.5 nM/5 nM) or a combination of drugs. Primary AML samples were incubated at 1 × 10^6^/ml in culture medium for four hours with Venetoclax (100 nM), AC220 (100 nM) or S63845 (25 nM/50 nM) or a combination of drugs. To measure the percentage of cells with loss of cytochrome C, cells were fixed in 2% para-formaldehyde following 4-hour drug incubation. Fixed and rinsed cells were permeabilised with saponin and labelled with Alexa-647-conjugated cytochrome C antibody. Leukaemic blast cells were identified using CD45 APC-H7 antibody (Becton Dickinson). LSCs were identified using CD34 PerCP antibody (Becton Dickinson) and CD38 (AT1) PE antibody (Beckton Dickinson). Data were collected on a FACSCanto II flow cytometer (Becton Dickinson) and analysed with FACS Diva software (Becton Dickinson).

### Western blot analysis

MV4-11 cells were treated for four hours with 10 nM Venetoclax, 5 nM S63845 or the drug combination. Cell lysates were prepared, separated by sodium dodecyl sulphate polyacrylamide gel electrophoresis, and transferred to nitrocellulose membranes. Detection antibodies included anti-MCL1 (S-19, sc-819) and anti-Bcl-2 (C-2) from Santa Cruz Biotechnology, Santa Cruz, CA, USA and anti-β-actin from Abcam, Cambridge, UK.

### Calculations and statistics

Fold excess additivism was calculated as a ratio of observed to expected values for drug combinations, where the expected value C is calculated from the Bliss algorithm for response to two compounds with effects A and B i.e. C= A + B −A*B [48]. This method allows for potentiation and augmentation as well as synergism. Statistical analysis was performed using the Statistical Package for Social Sciences, version 23 (SPSS, Chicago, IL, USA). P values of ≤0.05 were considered to represent significance.

## SUPPLEMENTARY MATERIALS FIGURE AND TABLE



## Abbreviations

AML: acute myeloid leukaemia
BAD: BCL-2 associated death promotor
BAK: BCL-2 homologous antagonist killer
BAX: BCL-2 associated X protein
BCL-2: B-cell lymphoma-2
BCL-XL: B cell lymphoma-extra-large
BID: BH3 interacting-domain death agonist
BIM: BCL-2 interacting mediator of cell death
CLL: chronic lymphocytic leukaemia
DNMT3A: DNA (cytosine-5)-methyltransferase 3A
FCS: foetal calf serum
FDA: Food and Drug Administration
FLT3: fms-related tyrosine kinase 3
FMS-ITD: fms-related tyrosine kinase 3 internal tandem duplication
IDH: isocitrate dehydrogenase
LSC: leukaemic stem cell
MCL-1: Myeloid Cell Leukaemia-1
NOXA: Phorbol-12myristate-13-acetate-induced protein 1
NPM1: nucleophosmin 1
RPMI: Roswell Park Memorial Institute
SPSS: Statistical Package for Social Sciences
STAT5: signal transducer and activator of transcription 5
