# Supplementary material for: Genetic biomarkers predict response to dual BCL-2 and MCL-1 targeting in acute myeloid leukaemia cells
Source: Oncotarget. 2018 Dec 28;9(102):37777–89. doi: 10.18632/oncotarget.26540 (PMC6340871; doi:10.18632/oncotarget.26540)
Supplement: Supplementary file 1 [file oncotarget-09-37777-s001.pdf]

Genetic biomarkers predict response to dual BCL-2 and MCL-1 targeting in acute myeloid leukaemia cells

SUPPLEMENTARY MATERIALS

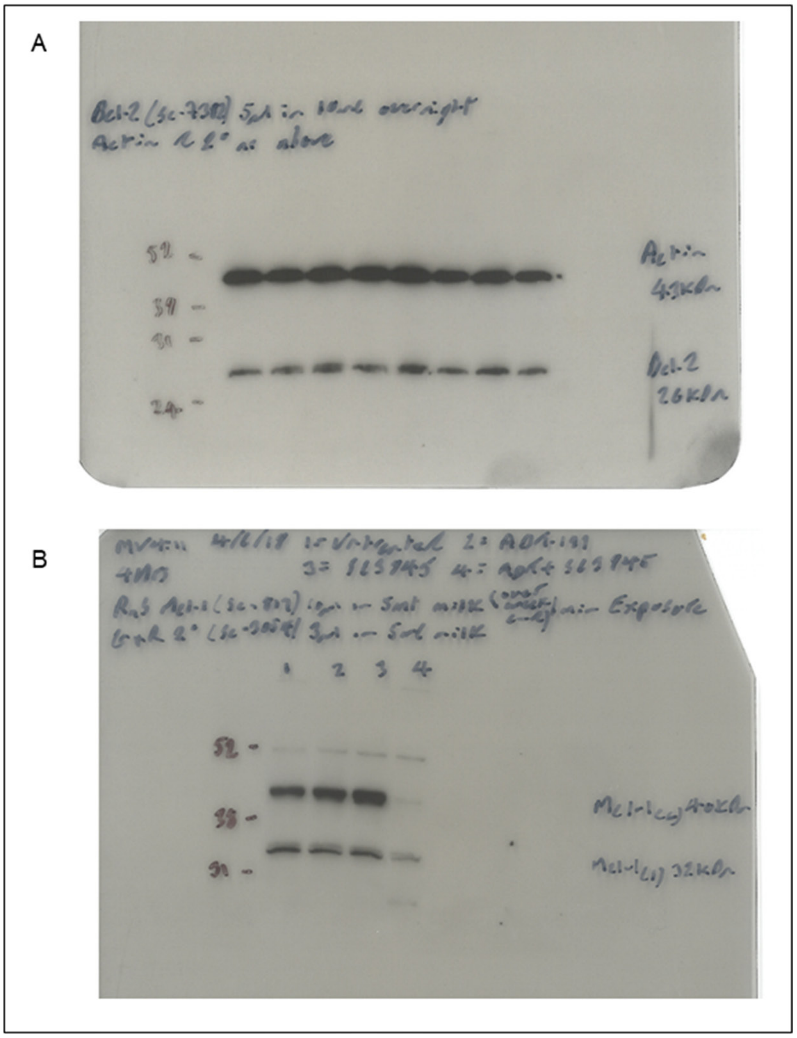

Supplementary Figure 1: Anti-apoptotic protein expression following treatment with the combination of Venetoclax and S63845. BCL-2 (Blot **A**) and MCL-1 (Blot **B**) protein was quantified in untreated MV4-11 cells (Lane 1) or cells treated for four hours with 10 nM Venetoclax (Lane 2), 5 nM S63845 (Lane 3) or the drug combination (Lane 4). The blots shown an example of two independent experiments.

Supplementary Table 1: Primer sequences

Primer	Sequence (5′–3′)
p53_exon5a_F	gtctccttcctcttcctacag
p53_exon5a_R	tgctcaccatcgctatctga
p53_exon5b_F	gcagctgtgggttgatt
p53_exon5b_R	agcaatcagtgaggaatcag
p53_exon6_F	ctgattcctcactgattgct
p53_exon6_R	taacccctcctcccagaga
p53_exon7_F	cttgggcctgtgttatctc
p53_exon7_R	agtgtgcagggtggcaagt
p53_exon8_F	cttcttgtcctgcttgctt
p53_exon8_R	tctcttttcctatcctgagtag
p53_exon9_F	cacctttccttgcctctttc
p53_exon9_R	ccccaattgcaggtaaaacag
IDH1_132_F	ttcagagaagccattatctgcaa
IDH2_132_R	cacattattgccaacatgacttactt
IDH2_140_F	tcctcacagagttcaagctgaag
IDH2_140_R	ggactaggcgtgggatgttt
IDH2_172_F	ggagcccatcatctgcaa
IDH2_172_R	ctccaccctggcctacct

Screening for p53 and IDH1/2 and mutations was via high resolution melting curve analysis with MeltDoctor HRM mastermix (Thermo Fisher Scientific, Loughborough UK) and performed on a 7500 Fast Real Time PCR system (Thermo Fisher Scientific) according to the manufacturer’s instructions. Supplementary Table 1 contains the primer sequences.
